# How to avoid describing your radiological research study incorrectly

**DOI:** 10.1007/s00330-020-06720-0

**Published:** 2020-02-21

**Authors:** Steve Halligan, Shedrack F. Kenis, Oshaani Abeyakoon, Andrew A. O. Plumb, Susan Mallett

**Affiliations:** 1 Centre for Medical Imaging, University College London UCL, Charles Bell House, 43-45 Foley Street, London, W1W 7TS UK; 2 Institute of Applied Health Sciences, University of Birmingham, Edgbaston, Birmingham, B15 2TT UK

**Keywords:** Clinical study, Clinical trial, Observational study, Pilot study, Feasibility studies

## Abstract

**Abstract:**

This review identifies and examines terms used to describe a radiological research “study” or “trial”. A taxonomy of clinical research descriptions is explained with reference to medical imaging examples. Because many descriptive terms have precise methodological implications, it is important that these terms are understood by readers and used correctly by researchers, so that the reader is not misled.

**Key Points:**

*• Multiple different terms are being used to describe radiological research “studies” and “trials”, and many of these terms have precise methodological implications.*

*• Radiological researchers sometimes use titles that describe their research incorrectly. This can mislead the reader as to what was actually done.*

*• It is important that readers and researchers understand the correct taxonomy of clinical research and that researchers adopt the correct description for their work.*

## Introduction

Understanding the methodological design used for an individual research study is pivotal because differing designs measure different outcomes, introduce different biases, and provide different levels of evidence. It is well-established, for example, that case-control studies generally provide less compelling evidence than randomised controlled trials and that retrospective designs are generally inferior to prospective [1]. However, researchers can confound this issue by inadvertently using misleading terms to describe their work. For example, a recent systematic review by the authors found that no radiological study had used the term “pilot study” appropriately [2]. This problem is not solely radiological; Arain and co-workers concluded that medical, “Pilot studies are still poorly reported, with inappropriate emphasis on hypothesis testing” [3]. Olsen and co-workers found that terms used to describe diagnostic test accuracy (DTA) studies sometimes lacked clarity [4]. As a basis for discussion on this issue, this review identifies methodological descriptions published in *European Radiology* and attempts to suggest appropriate terminology.

In preparation for this review, the first author searched the titles of all original research articles published in *European Radiology* 2015 to 2019 inclusive, encompassing volumes 25 to 29; five years. He noted all adjectives adjacent to the term “study”—identifying 68 different individual descriptions in total; many studies used multiple terms, for example, “single-centre retrospective observational study” [5]. Terms could be generally divided into five groups. One group described the imaging technology/modality investigated (9 broad terms), for example “a fMRI study” or “a radiographic study”. A group of just five terms described the subject studied: paediatric; animal; phantom; cadaveric; and twin. In 10 papers, “study” referred to an acronym, e.g. the “NELSON study” [6]. Six papers referred simply to “a study”, without further qualification. However, by far the largest group (47 individual terms) were terms that appeared to describe the methodological design used for the research (Table 1).

**Table 1:** Adjectives used in the title adjacent to “study” for articles published in *European Radiology* volumes 25 to 29 inclusive. Adjectives used three or more times over the period are shown in the Table. Adjectives used once or twice only are shown in the footnote.* Where multiple terms were used in a title, all were noted

Methodology descriptor	Frequency
Prospective	29
Retrospective	29
Feasibility	25
Multi-centre	22
Pilot	17
Preliminary	15
Comparative; comparison	14
Cohort; matched cohort; case-cohort	13
Case control; matched case-control	9
Observational	6
Single centre; single institution	6
Proof-of-concept	5
Ex-vivo; in-vivo; in vitro	6
Intra-individual	5
Propensity score matching	4
Diagnostic performance; diagnostic accuracy	4
Population based	3
Reader; independent reader; multi-reader – multi-case	3
Inter-observer; inter-rater; intra-reader	3
Longitudinal	3
Long-term	3
Safety; safety and efficacy	3

*Agreement; basic; case; clinical; clinically validated; controlled; dose-finding; dose-saving; experimental; exploratory; external validation; follow-up; global; histology validated; image quality; initial; international; large; non-inferiority; non-interventional; non-randomised; observer performance; patient; primary; prognostic panel; quantitative; rabbit model; randomised; spatial statistics; survey; reproducibility and reliability; validation

### Research study or trial? Prospective or retrospective?

Our search of *European Radiology* identified 269 articles whose title used the term “study”, whereas “trial” was used by just 34 articles over the same period. Overall, these studies and trials encompass a wide range of different objectives, including evaluating randomised controlled trials of radiological interventions, diagnostic test accuracy, evaluation of side effects, development of biomarkers, repeatability, interobserver agreement, development of imaging scores, imaging outcome measurements, evaluation of training, resource usage, and development of technical aspects of imaging procedures. While an acronym accounted for eight uses of “trial”, perhaps unsurprisingly “randomised controlled” was the commonest association. However, other descriptions included “randomised”, “randomised multicentre”, “prospective”, “screening”, and even “large” [7]. The authors would argue that “large” is a matter of opinion and it would be more informative to simply state the number analysed (or omit this information from the title altogether). We could find little definitive guidance regarding when to use the term “study” as opposed to “trial” but our understanding is that trial is typically used when participants receive interventions or tests prospectively, according to a research protocol.

**Fig. 1:**
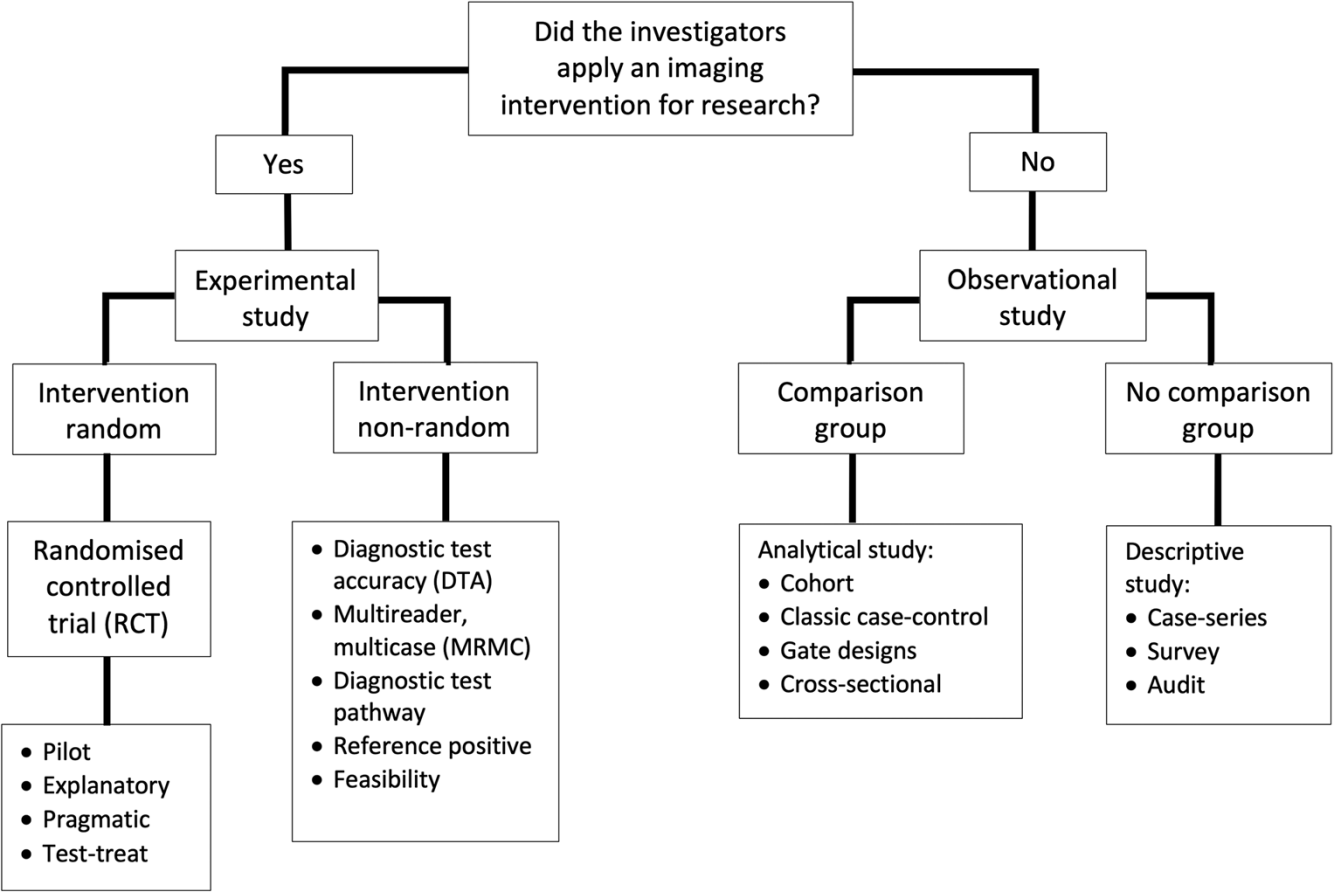
A taxonomy of clinical research designs for radiological research, adapted from Grimes and Schulz [8]

Figure 1 is a flowchart describing different types of clinical imaging research, adapted from Grimes and Schulz [8]. The pivotal decision is, “Did the investigators apply an imaging intervention or test?” If “yes”, it is clear that the investigators have performed an “experiment”; i.e. they likely have a hypothesis and have applied an intervention or test to evaluate this, a new MR sequence for example. Experiments that apply an intervention or test and then watch for results, such as improved diagnostic accuracy or patient outcomes, are “prospective” and “look forwards”. Prospective designs plan recruitment and data collection before patient characteristics, tests, or outcomes are measured. In contrast, retrospective studies “look backwards” and are usually termed “observational” studies because the intervention or test of interest was not originally applied with experimental intent. Our search of *European Radiology* found the terms “prospective” and retrospective” were the commonest used to describe studies, both applied 29 times each (Table 1). The highest level of evidence is based on protocol driven prospective studies that allow researchers to control for biases and to standardise and pre-specify study methods. The downside is that they are time and resource consuming. Retrospective studies are generally quicker and easier because the data exist already; they merely need compiling. Observational studies dominate the indexed literature for this reason [9], although are prone to bias, especially if there is no protocol for data recording; without a protocol, data may only be present for selected patients based on unreported criteria and may be collected using a mishmash of definitions and methods. It is self-evident that randomised trials are prospective so the description “prospective randomised trial” is unnecessary [10].

### Explanatory and pragmatic approaches to research

Randomised controlled trials (RCT) provide the highest level of research evidence because the intervention is assigned to the study population randomly, but the RCT does not guarantee impeccable results; the trial still needs to be well-designed etc. For example, the first author performed a RCT where patients were randomly allocated CT colonography or colonoscopy to diagnose colorectal cancer [11]. Such a RCT could be performed in a single, highly specialised centre, using expert radiologists and colonoscopists, and might randomise only those patients with very convincing symptoms (causing a spectrum bias towards patients with large, easy-to-diagnose tumours). We would then expect excellent results for CT but, unfortunately, the real world is not full of highly specialised hospitals bursting with experts, and patients’ symptoms vary. So, while such a trial might determine if CT can diagnose large cancers under “ideal” circumstances, a better reflection of day-to-day diagnostic accuracy would be achieved by performing the trial over multiple sites with more representative radiologists and consecutive accrual of all patients referred for testing. The first type of RCT is termed “explanatory” and determines diagnostic accuracy under ideal conditions, whereas the second is “pragmatic” and determines “real world” accuracy [12, 13]. Neither term was identified by the authors’ search of *European Radiology*. Importantly, it is increasingly recognised that the distinction between pragmatic and explanatory approaches is not restricted to RCTs but can be applied to DTA studies. Here, the relevant question for pragmatic radiological studies is how imaging tests influence day-to-day clinical decision-making [14].

The degree to which research findings extrapolate to similar patients in different centres and settings etc. is known as their “generalisability”, “transferability”, or “external validity” and is important because it is well-recognised that patients who participate in research may not be representative, and neither are centres who conduct research [15]. Indeed, within an individual RCT, it is good practice to maintain an “exclusion log” of patients who were potentially eligible but, for whatever reason, did not participate [16]. A comparison of eligible but excluded patients to those recruited may provide valuable evidence regarding applicability of findings to daily practice. Pragmatic research is more generalisable than explanatory. Whereas explanatory research is often single-centre, pragmatic research is usually multi-centre so as to enhance generalisability. We identified 22 instances where “multi-centre” was used to describe a study as opposed to 6 for “single centre”. However, this does not reflect the fact that multi-centre research is commoner; rather, it is rarer (because it is difficult) and the authors wish to broadcast the fact in their title.

### Non-randomised experimental designs: diagnostic test accuracy

RCTs tend to examine patient “outcomes” and are rare for imaging studies. Newer, “test-treatment” RCTs assign treatment contingent on the test result, for example randomising between test and no test and then only administering treatment to test positives [17]. However, non-randomised experimental designs are much commoner and tend to measure test accuracy (notably sensitivity and specificity) and focus on “results” rather than on “outcomes”. Indeed, the RCT design is inefficient and often impractical to assess DTA because participants receive just one test, and only participants where test results diverge contribute to the effective sample size. Accordingly, large numbers of participants are needed to achieve adequate statistical power. DTA studies can encompass multiple objectives beyond sensitivity and specificity, including evaluation of side effects, repeatability, interobserver agreement, development of imaging scores, imaging outcome measurements, evaluation of training, resource usage, and development of technical aspects of radiological procedures.

In DTA designs, a new test is evaluated against an independent standard test; i.e. participants receive both the new imaging test and a reference test [18]. The best reference is a true, independent “gold standard” but this can be difficult to achieve so researchers often evaluate against standard practice instead. This poses specific analytical challenges because a new test cannot, by definition, outperform the reference (i.e. the new test can never be better). Paradoxically, a new test that, in reality, outperforms the existing standard will appear worse after analysis. A solution might require a composite/consensus reference derived from multiple sources (and risking incorporation bias), or an outcome-based assessment, where the eventual clinical diagnosis defines whether the target pathology was present or not [19]. For example, the diagnostic “rate” of a cancer imaging test can be determined first (what proportion of cases did the new test diagnose compared with standard practice?) and then a cancer registry used to provide a definitive reference standard so sensitivity and specificity can be calculated after adequate follow-up. The obvious disadvantage is that this takes time. In “reference standard positive” designs [4], only those individuals testing positive by the reference standard receive the new test.

“Diagnostic test pathway” designs are increasingly popular and, like diagnostic accuracy studies, participants receive both intervention and standard tests [20, 21]. Results from the new test are revealed only when clinical decisions have been made and documented based on results from the standard test alone (i.e. reflecting standard clinical practice in the absence of the new test): By then revealing results from the new test, it is possible to determine how the new test impacts on patients’ clinical trajectory [20]. More advanced designs compare multiple tests, with all evaluated against a reference. Where the same participants receive all the tests being compared as well as the reference standard, then biases due to differences in participants, study design, and methods are minimised providing a higher level of evidence for a test comparison [22]. Healthcare providers are increasingly interested in not only diagnostic accuracy but also how different trajectories following a test result consume evermore scarce resource. For example, in the UK, the National Institute for health and Clinical Excellence (NICE) Diagnostics Advisory Committee recommends tests for adoption not only on the basis of diagnostic accuracy but also on whether they are cost-effective.

Very surprisingly, the terms “diagnostic performance” or “diagnostic accuracy” were used rarely to describe a study (Table 1). The terms “comparative/comparison” were used 14 times but these terms are used across a range of research objectives and designs, and authors must take care because “comparison” is often misused in diagnostic accuracy designs. The term “intra-individual” was encountered 5 times. We would expect it to be used frequently in studies that compare different imaging methods within the same participant. We also identified terms that applied to the observer, for example “reader”, “independent reader”, and those that supplied further detail regarding the design such as “inter-observer”, “inter-rater”, and “intra-reader”. A well-recognised and efficient design is for multiple readers to read multiple cases, termed “multi-reader multi-case” (MRMC) studies and used frequently in radiological research [23], but, surprisingly, just one article used this term [24].

### Feasibility and pilot studies

There terms “feasibility”, “preliminary”, “pilot”, and “initial” study were used 25, 17, 15, and once respectively (Table 1). Indeed, one study used two terms together, a “pilot feasibility study” [25]. While these terms appear interchangeable, they are not. Feasibility studies are research that asks, “can this study be done?” They aim to improve the precision of uncertain parameters, often to inform decisions regarding funding subsequent research [26]. For example, research often fails because anticipated recruitment is over-optimistic. A feasibility study might therefore assess whether adequate numbers of potentially eligible patients can be identified and/or the proportion willing to participate, thereby preventing larger studies that are doomed to fail. Such studies might investigate a range of potential outcome measures in order to determine which is most appropriate for a larger definitive study (for example, because it can be measured precisely and/or is not burdensome to acquire). Crucially, feasibility studies are not powered to investigate the primary outcome of a proposed substantive study—if they were, then a subsequent larger study would be unnecessary. Although almost always prospective, a feasibility study could potentially be retrospective if the necessary data exist already, for example the number of patients with a specific disease presenting to a hospital annually.

Pilot studies are similar to feasibility studies but differ in that they are a fully worked-up but “miniature” version of a planned subsequent main study, i.e. a “small-scale test of the methods and procedures to be used on a larger scale” [27]. A pilot study might follow a feasibility study if the former suggests that sufficient patients can be identified and the primary and secondary outcomes measured with reasonable precision, for example by testing the ease with which study proformas can be completed and retained, or by investigating whether the methods developed via a feasibility study are transferrable to other centres. Pilot studies therefore play a pivotal role in mitigating unwanted surprises when performing large trials/studies and can save considerable time and expense. The UK Medical Research Council guidance on designing and evaluating complex interventions recommends that pilot studies be conducted before any definitive large-scale evaluation [28]. A common scenario involves a single-centre pilot study followed (if successful) by a definitive multi-centre study to recruit sufficient participants necessary to analyse the primary outcome.

Pilot studies are always prospective so the description “prospective pilot study” is unnecessary [29]. Pilot studies measure more factors than feasibility studies and, similarly, are not powered around the definitive primary outcome, for example diagnostic accuracy, because as we have said, a larger study would then be unnecessary. Nevertheless, a recent systematic review found that 27% of radiological studies described as “pilots” were solely retrospective and 85% reported underpowered metrics of diagnostic accuracy [2]. The term “pilot” is not an effective disguise for a small, underpowered study. Because pilot studies are miniature substantive studies, it follows that the data they collect may potentially contribute to the subsequent study and its final analysis, in which case they are termed “internal” pilots. Alternatively, if pilot data are analysed in an unblinded fashion, and/or methodological modifications during the pilot render the data unsuitable for incorporation into the final analysis, then “external” pilot is appropriate (neither term “internal” or “external” was identified by our search).

It is increasingly common for funders to require a pilot before committing to a substantive study. Progression is terminated if the pilot is unsuccessful. In our experience, it is common for researchers to define success imprecisely. The correct approach is to pre-specify precise and unambiguous “stop-go” criteria. Such criteria commonly revolve around a minimum number of subjects recruited over a specified time-scale. It is also worth considering the fact that only patients with the primary outcome of interest provide useful data; a pilot study recruiting 100 subjects that aims to diagnose cancer is useless if no patient has the disease. Recommendations to estimate pilot sample size are available, for example using a confidence interval around the anticipated standard deviation [30], or 3% the anticipated size of the substantive trial [31].

“Proof of concept” was used by five papers (Table 1), which appeared to imply method development. The authors believe the terms “preliminary” and “initial” are meaningless. Preliminary to what? If preliminary to a substantive study, then the appropriate terms are “feasibility” or “pilot” depending on the context. Like “pilot” and “feasibility”, “preliminary” and “initial” do not excuse underpowered, poorly performed research. Indeed, it has been argued frequently that reporting underpowered research is unethical because such studies encourage clinical practice based on invalid results [32–34]. English will not be the first language of many researchers and this may contribute to some misunderstanding around the precise meaning of some terminology. Nevertheless, English is the language used generally and researchers are therefore obliged to use it correctly.

### Observational studies

Our search identified the term “observational” just six times (Table 1), despite the fact that such studies dominate published research [8]. Observational studies differ from experimental studies in that no test or intervention is applied by researchers, who instead simply “observe” events. Such studies can be divided on the basis of whether there is a comparison group (exposure group and control group) or not (Fig. 1). “Analytical” studies incorporate a comparison group and are classified depending on the timing of patient recruitment relative to reference standard diagnosis or outcome measurement. In general, observational studies suffer from the fact that exposure is not randomly assigned but is observed in the data, so it may be confounded, for example, with patient characteristics. This may bias conclusions regarding relationships between exposure and outcomes. Observational studies without any comparison group are often case-series, audits, or surveys. The case report of a single subject lies at the bottom of the research hierarchy [8].

### Study timeframe

An important aspect of study design is whether patient recruitment or selection into the study precedes measurement of the study outcome or reference standard. Cohort studies “march towards outcomes” [35] and are similar to experimental studies in that an outcome or reference standard is measured after exposure; our search identified the term “cohort” 13 times. An example would be a cohort study that did not administer any intervention, but prospectively identified patients who did and did not receive gadolinium agents and then waited to follow up these patients to look for subsequent brain deposition. Cohort studies are usually prospective (and are thus time-consuming, expensive, and most efficient for common outcomes) but can be retrospective where patients have been recruited prospectively for another purpose.

In contrast, case-control studies have been described as “research in reverse” [36] because the outcome is measured first and the researchers then “look backwards” to see if the participant encountered the exposure of interest. For example, researchers have identified patients with high femoral bone gadolinium and then searched for patients with prior MRI exposure [37]. We identified the term “case-control” 9 times. Case-control studies are usually retrospective and, as such, are relatively inexpensive and quick, and efficient for rarer outcomes, especially those that take time to develop. However, they are prone to considerable recall and selection bias and often do not include a representative spectrum of patients by just including clear cases and controls (more so than cohort studies where researchers measure at the time of exposure). Case-control studies have no clinically relevant denominator as this is determined by the study design, unlike cohort studies which can be used to calculate incidence rates, relative risk, etc. Nested case-control designs (based on selection of cases and controls from a prospectively recruited patient cohort) are often used in radiological studies to enrich for patients whose disease has low prevalence. These studies can be designed to have low risk of bias if cases and controls are randomly selected from these groups in the cohort. Methodologists are increasingly substituting “case-control” terminology with “two gate design” or “one gate design” to indicate whether two separate sets of eligibility criteria are used (classic case control) or one set of eligibility (nested case control where eligibility is based on the cohort criteria) [38].

“Cross-sectional” studies (often used in “frequency” or “prevalence” or diagnostic studies) are a “snapshot in time” [8], because exposure and outcome are measured simultaneously. An example from *European Radiology* is a study that examined symptoms (outcome) in patients with pulmonary nodules (exposure) [39].

### Reporting guidelines

Research reporting guidelines have exploded over recent years and strive to ensure that published work can be understood by readers, replicated by other researchers, used for clinical decision-making, and extracted for systematic review. Guidelines are simple, structured tools used by researchers when reporting their work. Adherence ensures that the report contains the “minimum dataset” of information necessary to achieve these aims. The EQUATOR (Enhancing the QUAlity and Transparency Of health Research) network assembles reporting guidelines for major study types [40]. STARD [18] is one such guideline familiar to radiologists, applicable to DTA studies. STARD item 1 states that the title should, “identify as a study of diagnostic accuracy using at least one measure of accuracy” [18]. QAREL [41] is a tool used to assess the quality of DTA studies, for example when performing a systematic review.

### Summary

Of course, much radiological research does not manifest as a “study” or trial”. Predictive biomarker research and prognostic model development are obvious examples, and methodological issues with these are well-recognised also, especially around sensible validation [42–44]. Also, our review was far from exhaustive: We identified adjectives immediately adjacent to the term “study” so will have missed methodology descriptions when elsewhere in the title. Nor is this Editorial seeking to criticise individual researchers; the authors themselves have been guilty in the past and are part of the wider research community who aim to constantly improve research reporting. We simply wish to illustrate the general point that multiple terms are used to describe research in the face of an existing practical taxonomy for clinical research. Noting the absence of a generally agreed taxonomy that applies to diagnostic research [4], nevertheless, it is important to avoid terms that are not in widespread use or are misleading. Moreover, when using a well-recognised term, it is important it is used correctly and reflects what was actually done. It has been suggested that radiological research is methodologically weaker than other medical disciplines [45, 46]. We hope that our brief review helps alleviate this via a clearer understanding of different study descriptions and their specific implications. It is also interesting to note that most clinical research articles published in *European Radiology* did not specifically indicate a study design in their title at all. Authors should consider doing this, so that readers are immediately aware of the research design used and the likely evidence level provided by the study.

## Abbreviations

CT: Computed tomography
DTA: Diagnostic test accuracy
MRMC: Multi-reader, multi-case
MRI: Magnetic resonance imaging
NICE: National Institute for health and Clinical Excellence
RCT: Randomised controlled trial
